# Treatment Options for Motor Fluctuations in Parkinson's Disease

**DOI:** 10.1002/mds.70107

**Published:** 2025-10-28

**Authors:** Günter Höglinger, Paul Lingor, Matthias Höllerhage, Claudia Trenkwalder

**Affiliations:** ^1^ Department of Neurology LMU University Hospital, Ludwig‐Maximilians‐Universität (LMU) München Munich Germany; ^2^ Munich Cluster for Systems Neurology (SyNergy) Munich Germany; ^3^ German Center for Neurodegenerative Diseases (DZNE) Munich Munich Germany; ^4^ Department of Neurology TUM Klinikum Rechts Der Isar Munich Germany; ^5^ Department of Neurology Hannover Medical School Hanover Germany; ^6^ Paracelsus‐Elena‐Klinik Kassel Germany

**Keywords:** Parkinson's disease, fluctuations, therapy, on‐demand therapies

We read with interest the systematic review on treatment options for Parkinson's disease (PD) motor fluctuations, published as an International Parkinson and Movement Disorder Society (MDS) evidence‐based medicine (EBM) review article.^1^

We appreciate the authors' motivation to provide guidance in determining which therapeutic options are safe and efficacious to reduce patients' disease burden. The article appears intrinsically consistent, following the strict Grading of Recommendations Assessment, Development and Evaluation (GRADE)‐based methodology.

Having recently evaluated the literature on the same subject while developing the German Society of Neurology Guidelines for PD,^2^, ^3^ we wish to share some comments on this article.^1^

First, rapid‐release (dispersible/soluble) oral levodopa is still among the most frequently prescribed off‐rescue medication. This option is not mentioned in the MDS review.^1^ In the absence of randomized controlled trials to demonstrate superiority over other therapeutic options, there is still robust clinical experience supporting this safe and efficacious option.

Inhaled levodopa is not mentioned either in the MDS review,^1^ although the criteria set forth by the authors appear to qualify consideration of the pivotal study demonstrating its efficacy to significantly reduce motor fluctuations.^4^ It might have been omitted since daily off‐time was not the primary readout. The drug is still approved for this indication by the US Food and Drug Administration (FDA) and the European Medicines Agency (EMA).

Likewise, the commonly used dopamine agonist piribedil is not mentioned.^1^

Intermittent application of apomorphine, either subcutaneous or sublingual, has been declassified by “insufficient evidence” in the MDS review,^1^ although published evidence may suggest otherwise^5^, ^6^ and these treatment options are approved, reimbursed, and administered successfully in many countries.

The classification of istradefylline as “likely efficacious” is interesting, since the EMA issued a negative opinion in 2021,^7^ refusing approval for istradefylline as an add‐on treatment for motor fluctuations, considering the available evidence as inconsistent and not satisfactorily showing effectiveness to reduce ‘off’ time. A pooled analysis of eight phase IIb/III trials suggested that istradefylline reduces daily off‐time by only 0.38 or 0.45 hr more than placebo at 20 or 40 mg/day, respectively.^8^

These discrepancies between the conclusions reached in this article^1^ and the current options approved and available to aid patients with motor fluctuations demonstrate the striking limitations of the GRADE methodology, when stringently applied. Obviously, there is no possibility to consider other than the specified endpoints, relative effect sizes, or clinical experience in the absence of formal data for “old” drugs, and regional availabilities.

The article also does not comment on the specific medical conditions in which the different options might be advantageous for individual patients (eg, continuous vs. on‐demand medications, enteral vs. parenteral administration, different half‐lives, renal or hepatic elimination, and so on). These aspects, however, are very important when making treatment decisions. Furthermore, the choice of options should also consider individual patient's side effect profiles, which in reality often become decisive factors.

Thus, the strict EBM methodology apparently may deliver conclusions that are strikingly distinct from the options available and meaningful in reality for patients and caring neurologists in different regions. Therefore, a reconsideration of the EBM methodology used by the MDS Task Forces to provide true guidance for clinical practice may be warranted.

## Author Roles

(1) Research Project: A. Conception, B. Organization, C. Execution; (2) Statistical Analysis: A. Design, B. Execution, C. Review and Critique; (3) Manuscript: A. Writing of the First Draft, B. Review and Critique.

G.H.: 3A, 3B.

P.L.: 3B.

M.H.: 3B.

C.T.: 3B.

## Financial Disclosures for the Previous 12 Months

G.H. participated in industry‐sponsored research projects from AbbVie, Biogen, Biohaven, Novartis, Roche, Sanofi, and UCB; has ongoing research collaborations with AbbVie, Ferrer, Roche, and UCB; serves as a consultant for AbbVie, Alzprotect, Amylyx, Bayer, Bial, Biogen, Biohaven, Ferrer, Lundbeck, Merz, Novartis, Roche, Sanofi, Takeda, TEVA, and UCB; and received honoraria for scientific presentations from AbbVie, Bayer, Bial, Biogen, Merz, Roche, TEVA, UCB, and Zambon. P.L. participated in industry‐sponsored research projects from Alexion, Biogen, Blue Rock Therapeutics, Corcept Therapeutics, Mitsubishi Tanabe, Novartis, Theranexus, and Trace Neuroscience; serves as a consultant for AbbVie, Novartis, ITF Pharma, Raya Therapeutics, Stadapharm, Trace Neuroscience, Woolsey Pharmaceuticals, and Zambon; and received honoraria for scientific presentations from Bial, Desitin, ITF Pharma, and Zambon. M.H. participated in industry‐sponsored research projects from AbbVie, Biogen, and Roche; serves as a consultant for AbbVie and Teitur Trophics; and received honoraria for scientific presentations from AbbVie and DESITIN. C.T. served as consultant for AbbVie, Roche, Boehringer, UCB, Bial, and Convatec; and has received honoraria for lectures from AbbVie, Bial, Esteve, and STADA.

## Data Availability

The data that support the findings of this study are available on request from the corresponding author. The data are not publicly available due to privacy or ethical restrictions.
